# Thrombosis Models: An Overview of Common In Vivo and In Vitro Models of Thrombosis

**DOI:** 10.3390/ijms24032569

**Published:** 2023-01-29

**Authors:** Sana Ayyoub, Ramon Orriols, Eduardo Oliver, Olga Tura Ceide

**Affiliations:** 1 Department of Pulmonary Medicine, Dr. Josep Trueta University Hospital de Girona, Santa Caterina Hospital de Salt and the Girona Biomedical Research Institute (IDIBGI), 17190 Girona, Spain; 2 Centro de Investigaciones Biologicas Margarita Salas (CIB-CSIC), 28040 Madrid, Spain; 3 Centro de Investigación en Red de Enfermedades Cardiovasculares (CIBERCV), 28029 Madrid, Spain; 4 Biomedical Research Networking Centre on Respiratory Diseases (CIBERES), 28029 Madrid, Spain

**Keywords:** animal thrombosis models, endothelial dysfunction, hypercoagulation, in vivo thrombosis models, stasis, stenosis, thrombosis

## Abstract

Occlusions in the blood vessels caused by blood clots, referred to as thrombosis, and the subsequent outcomes are leading causes of morbidity and mortality worldwide. In vitro and in vivo models of thrombosis have advanced our understanding of the complex pathways involved in its development and allowed the evaluation of different therapeutic approaches for its management. This review summarizes different commonly used approaches to induce thrombosis in vivo and in vitro, without detailing the protocols for each technique or the mechanism of thrombus development. For ease of flow, a schematic illustration of the models mentioned in the review is shown below. Considering the number of available approaches, we emphasize the importance of standardizing thrombosis models in research per study aim and application, as different pathophysiological mechanisms are involved in each model, and they exert varying responses to the same carried tests. For the time being, the selection of the appropriate model depends on several factors, including the available settings and research facilities, the aim of the research and its application, and the researchers’ experience and ability to perform surgical interventions if needed.

## 1. Introduction

Thrombosis refers to pathological clot formation within the blood vasculature that may limit or block the blood flow. This may lead to severe conditions such as stroke, pulmonary embolism (PE), myocardial infarction (MI), organ and tissue ischemia, or other conditions depending on the site where the clot has formed. This disorder is among the leading causes of morbidity and mortality worldwide, as it was estimated to account for one in four deaths in 2010 [1]. Stroke and MI are among the most threatening thrombotic incidents [2]. Decreased quality of life, poor prognosis, and shorter life expectancy are associated with several thrombotic-related disorders, like chronic thromboembolic pulmonary hypertension (CTEPH), which occurs due to obstruction in major arteries that supply the lungs and is considered a long-term complication of PE [3,4].

Several factors that affect normal coagulation and homeostasis contribute to thrombosis, which may be acquired, inherited, or a combination of both. Homeostasis imbalance due to endothelial defects, changes in the blood flow, or alternation in fibrinolytic and coagulation components leading to a hypercoagulable state all contribute to thrombosis development [5].

Animal models of thrombosis are critical in vascular research for understanding the complex mechanism of hemostasis and thrombus formation and for anti-thrombotic drug screening. Several in vitro and in vivo models are routinely used in research involving thrombosis, which will be summarized in this review focusing on the most commonly used models.

## 2. Thrombosis Overview

The single-sheet endothelial layer lining the blood vessels acts as a barrier between the blood’s components and the underlying layers with their highly reactive elements [6]. A complex mechanism that involves an interaction between the endothelial layer, blood components, inflammatory factors, cytokines, coagulation factors, and plasma proteins is responsible for maintaining a normal blood flow within the vasculature. An imbalance between any of these components promotes a hypercoagulable state and thrombosis development [7].

The two main types of thrombosis are venous thrombosis and arterial thrombosis, which develop in the veins and arteries, respectively. Both types occur through a distinctive pathological mechanism. On one side, venous thrombosis has been associated with dysfunction in the endothelium and activation of the clotting system and the thrombus is referred to as red thrombus, being rich in blood cells; whereas arterial thrombosis is linked with platelet activation and is known as white thrombus [8,9,10]. However, they might share some common pathophysiological pathways and risk factors [11].

The treatment and management of thrombosis are different depending on many factors, including age, history, and underlying comorbidities, among others, but most importantly, they depend on the type of thrombosis. For instance, anticoagulants are mainly used for the management of venous thrombosis, while antiplatelet agents are used for arterial thrombosis. Treatment guidelines for the management of the different types and subtypes of thrombosis are constantly updated for appropriate control of this complicated disorder [12,13,14].

## 3. Methods and Selection Criteria

The main search term that was used to find the studies included in this review is ‘thrombosis models’, and this was combined with other words, including in vitro, in vivo, murine, porcine, swine, pigs, rodents, rats, mice, zebrafish, stasis, stenosis, hypercoagulation, endothelial injury, shunt model, thrombosis on a chip, microfluidics, and flow chamber. Studies in this review were included from all years; no restriction was placed on publication dates. For in vivo models, studies on animals other than mice, rats, pigs, and zebrafish were excluded.

## 4. Models for Thrombosis

### 4.1. In Vitro Models

Reproducing a vascular disorder outside the biological system is a complicated procedure, which usually requires microfluidic devices to mimic the biological system. Traditional in vitro models mostly evaluate a single aspect or component of the vascular disorder, like the platelet aggregation assay, which mainly evaluates the influence of agonists or antagonists on the platelets’ functions [15].

Among the earliest in vitro thrombosis models was the capillary thrombometer, described by Morawitz and Jürgens in 1930, which consisted of a horizontal glass capillary connected to two glass columns and was used to assess in vitro thrombus formation rate through the movement of blood back and forth in the glass capillary until the thrombus was formed [16].

On the other hand, the more recent thrombosis in vitro models, like thrombus on a chip, include more components, with better-controlled conditions to accurately mimic the biological system, including the vascular structure, blood cellular components, and signaling molecules.

#### 4.1.1. Macrofluidic- and Microfluidic-Based Models

**Figure 1:**
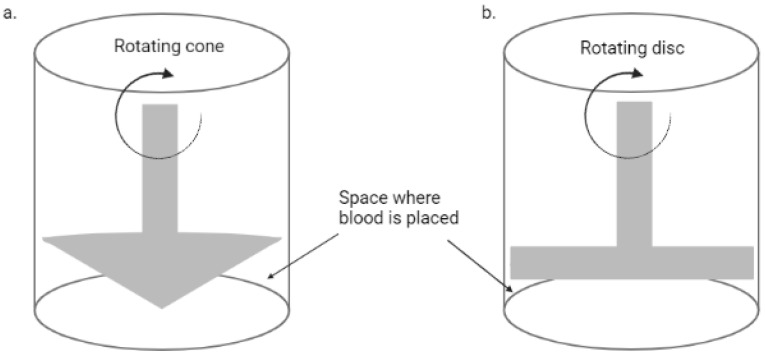
A simplified diagram of a rotational rheometer. (**a**) Cone and plate rheometer; (**b**) two-disc rheometer (Created with BioRender.com).

The emergence of microfluidics technology has allowed the development of in vitro models of the vascular system with precise manipulation of the blood flow and cellular components, also providing a major advantage of using smaller blood volume as compared to earlier methods. Before this technology, macrofluidic systems were used, including a cone-and-plate device and two-disc rheometer, as shown in Figure 1, which rely on the rotation of the cone or disc for shear stress induction, and have been used to evaluate the influence of varying shear stress on endothelial cells [17]. The cone-and-plate device was the first to report that endothelial cells’ function and morphology are modulated by altered shear stresses [18]; however, they are not as common nowadays as the microfluidic systems.

A well-known model that was developed with the microfluidics technology is the ‘thrombosis on a chip’, which allowed the modification of the endothelial surface to mimic different prothrombic conditions, easily introducing foreign agents for study, and high-throughput drug screening, among many other applications [19,20].

Clearly, the design of microfluidic systems to study blood vascular disorders has gone through several developments throughout the years, from the use of glass capillaries to the three-dimensional endothelial cells lined with hydrogels to better mimic the biological system [21]. Mentioned below are the most used devices in research nowadays, which rely on macro- and microfluidics technologies.

##### Flow Chambers

This is among the earliest methods used to model thrombosis in vitro, which generally represents a channel which blood passes through. This device has gone through many modifications throughout time and is commonly used for quantitative assays to measure in vitro thrombus formation [22].

Different flow chamber-based devices have been developed, ranging from simple to more complicated systems. These devices vary in size and flow surface coatings, which include endothelial cells, collagen, or other synthetic peptides, and are applied mainly to assess platelets’ function and hemostasis [22,23]. Several devices are classified under this technology, including viscometers, parallel-plate, annular, and tubular flow chambers, some of which have a constant flow of fluid while others have a controlled flow rate [24]. Also, custom-made devices are developed by a number of laboratories relying on this method [25].

**Figure 2:**
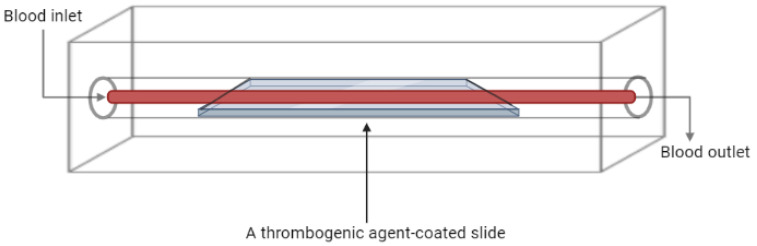
Schematic diagram of a flow chamber-based thrombosis model (Created with BioRender.com).

A common approach in designing a flow chamber-based thrombosis model is the perfusion of blood sample over a surface coated with a platelet-activating or thrombogenic agent, as shown in Figure 2, in which collagen is the most commonly used. However, other agents like fibrin, fibrinogen, von Willebrand factor (vWF), and others are also employed [26,27]. Generally, the method involves coating a glass coverslip with the thrombogenic agent, followed by blood perfusion over the coverslip under controlled shear rate, and in some cases the process is monitored in real time [28,29]. Some studies reported the use of cell-free homogenate of atherosclerotic plaque as a thrombogenic agent and were reported to generate in vitro thrombosis through direct activation of platelets; thus, this method can be used as a model for arterial thrombosis and to evaluate novel anti-thrombogenic agents [29,30].

Considering the variations among the designed devices employing this technology, some initiatives have been undertaken to standardize these flow-based assays; recommendations have been given regarding standardizing the flow chambers and a cost-efficacy comparison has also been considered [22]. This is an important step in this area and, if adopted by the designing companies, will further enhance reproducibility in the carried tests.

##### Thrombosis on a Chip

These models also employ the same concept as flow chambers but can be considered unique in terms of the ‘chip’ feature. These chips can be designed using soft lithography or bioprinting technology where microchannels are created using different materials and can be functionalized with endothelial cells [31].

**Figure 3:**
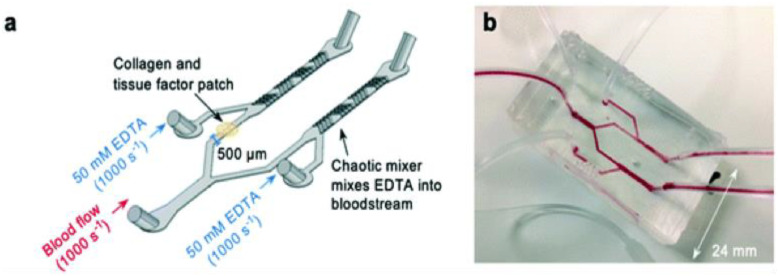
Novel thrombosis-on-a-chip device to measure occlusion time. (**a**) A schematic illustration of the ‘EDTA-quenched’ device; (**b**) a photograph of the device. Reproduced from Ref. [33] with permission from the Royal Society of Chemistry.

Figure 3 shows a thrombosis-on-a-chip model that employs a novel method to develop occlusive thrombosis and can be used to evaluate the inhibition of thrombus formation by different compounds. In this device, a bifurcation system is designed where blood enters through a single inlet, passes through two different branches, and leaves from two outlets, which is believed to induce the formation of occlusive thrombus [32]. In one channel, a patch of collagen and tissue factor (TF) is placed to mimic plaque rupture. Two additional inlets linked to each arm of the device were also designed to incorporate ethylenediaminetetraacetic acid (EDTA) downstream of the collagen and TF patch, which was used to assess the efficacy of an antiplatelet drug [33].

The application of this system in thrombosis includes high-throughput screening of anti-thrombotic drugs, the assessment of changes in different cellular components under different conditions, like shear stress and/or testing chemical reagents, and replacement of standard clinical tests for clotting and hemostasis assessments [34,35], as human blood can be used in these devices in very small amounts.

The main disadvantage of this microfluidic-based system is that the small device size does not allow the exact recreation of the pathological conditions, especially regarding altering blood flow over a longer distance, which is related to many thrombotic pathological conditions [36], since in these devices the blood moves a very short distance that does not recapitulate the human condition.

##### Other Microfluidic-Based Models

Considering the pathophysiology of venous thrombosis and the time it usually takes to develop, in vitro venous thrombosis models are not as common as arterial thrombosis. However, some studies reported the development of in vitro models of venous thrombosis.

In one study, a microfluidic device with geometries similar to human venous valves was designed, employing primary and secondary vortex characterized by the point of vortical flow and the point of low shear rate, respectively, that are believed to mimic human conditions and support thrombus formation [37,38]. The study reported in vitro venous thrombosis development through a three-step process, including initial fibrin formation at the site of the secondary vortex, followed by platelet delivery at the site between the primary vortex and the formed fibrin with the aid of red blood cells, and then platelet adherence to the fibrin, resulting in thrombus growth and escape to the bulk flow [39]. This device is among the few in vitro thrombosis models that recapitulate venous thrombosis.

Some in vitro models were created to recapitulate shear stress induced by blood-contacting medical devices. This is an important issue that has been characterized and addressed by Chen et al., 2015, using a novel blood-shearing device designed to mimic the pathological flow conditions of high shear stress and short exposure time caused by medical devices like catheters, stents, heart valves, and many others, helping in understanding the tendency and mechanism of developing thrombosis in patients implanted with these devices [40,41,42].

A list of some in vitro thrombosis models is summarized in the Table 1 below.

### 4.2. In Vivo Models

In vivo thrombosis models have been developed using various techniques that relied on the Virchow’s triad, which describes the three main elements involved in thrombus formation, which are damage to the vascular wall, disturbance in blood flow, and the presence of a hypercoagulable state [55]. Different methods can be applied to initiate any of these conditions, like promoting coagulation through the use of certain chemicals, directly injuring the vasculature to damage the endothelium, or artificially inducing stenosis to alter blood flow, among different other methods [56,57].

This review focuses on the animal models that are most used in research, including murine models, porcine models, and zebrafish.

#### 4.2.1. Murine Models

Rats and mice are among the most common animal models that are used to recapitulate different human disorders; being mammals means they share some common physiological processes with humans. They also share similarities with human genetics, as almost every disease-associated gene in humans has a counterpart in rats and mice [36]. They also provide numerous opportunities for genetic manipulation, and their small size allows ease of handling for maintenance and performing of different tests.

As previously mentioned, in vivo thrombus development involves the creation of endothelial injury, promoting a local hypercoagulable state, or artificial induction of vascular stasis or stenosis, and all these parameters have been employed in murine thrombosis models either alone or in combination with each other using different techniques, as described below.

##### Induction of Endothelial Injury

This can be done with the use of laser- or photochemical-induced injuries, where a beam of laser is focused on a blood vessel that is mostly but not always exposed through surgery. In photochemical-induced injury, the model is first treated with a chemical, like the commonly used Rose Bengal, followed by subsequent light illumination to further promote endothelial damages [58]. In these cases, the developed thrombus can be monitored over time using intravital microscopy and fluorophores that specifically target the thrombus [59]. On the other hand, direct mechanical injuries can also be induced by scrapping the endothelial wall using forceps to pinch a surgically exposed blood vessel [60], or through excision and ligation [61] or any other method that physically damages the endothelium.

Chemicals like ferric chloride (FeCl_3_) are commonly used and have been proven to induce occlusive thrombus in a dose-dependent manner, where they can be applied to surgically expose vessels to promote endothelial injuries via free radical generation and induction of oxidative stress [62,63].

##### Promoting Hypercoagulation

Factors that promote hypercoagulation are normally used in combination with other methods, like stasis, stenosis, or endothelial injury, to accelerate thrombus development. Serum, tissue factor, and high-fat diet (HFD) are among the common agents that are used to promote coagulation.

Hyperlipidemia is known to induce a hypercoagulable state by promoting several coagulation factors and fibrin deposition on the vascular wall, and the state is shown to be reversed when the high fat is withdrawn from the diet [57]. When combined with arteriovenous (AV) shunt models or ferric chloride-treated models, diabetic fatty rats were shown to develop thrombosis at a faster rate [64].

Serum and tissue factor are also commonly used as hypercoagulable agents in animal models, where the administration of serum is usually accompanied with vascular ligation to facilitate thrombus formation. One study reported that heterologous serum obtained from human blood samples had a stronger thrombogenic potential in rats than homologous serum [65]. Alternating the serum components can help elucidate the mechanism of thrombus development and the factors involved in thrombogenicity [66]. On the other hand, tissue factor, which is normally a glycoprotein that is expressed at the site of vascular injury, is used to induce thrombosis by direct infusion into the blood vessel [65,67,68].

Also, any antifibrinolytic agents that inhibit fibrin degradation can be employed to promote hypercoagulation, like tranexamic acid, which acts by binding to the surface of plasminogen or plasmin, preventing it from binding to and degrading fibrin, and was used to induce acute hypercoagulation in rat models by a single intragastric administration [69].

Transgenic murine models with hypercoagulable states are also commonly employed in thrombosis studies. In this regard, one study reported that transgenic mice with altered thrombomodulin (TM) gene, that plays a role in anticoagulation, referred to as the TM^Pro/Pro^ model, has an increased vascular fibrin deposition and a higher tendency to develop thrombosis when endothelial injury or stasis is induced by FeCl_3_ treatment or arterial ligation, respectively [70]. A review on the available transgenic mouse models of venous thrombosis has been reported by Audrey et al., 2007 [71].

##### Induction of Stasis or Stenosis

This method is mostly employed in larger vessels and refers to complete or partial blockage of blood flow, which is most commonly accomplished by narrowing the vessels by tying them with a ligature or compressing with a forceps; the method was first introduced in 1976 [72], and is referred to as Folt’s model. One study applied vascular ligation in mice of different ages and found that older mice have a vascular environment that promotes thrombus development [73].

**Figure 4:**
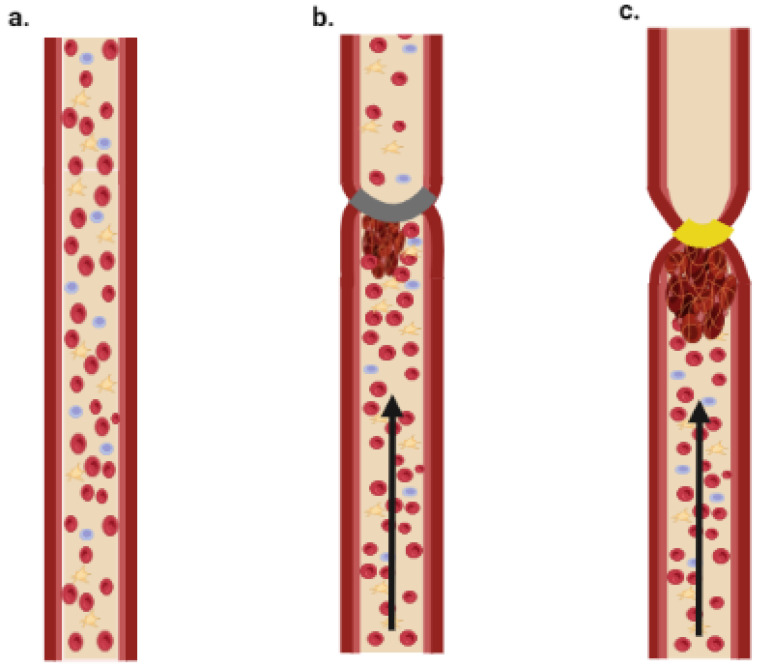
Schematic illustration of stasis and stenosis models. (**a**) Normal blood flow; (**b**) stenosis model—thrombus is formed due to partial block of blood flow; (**c**) stasis model—thrombus is formed due to complete block of blood flow (Created with BioRender.com).

Figure 4 shows a simplified diagram of stasis and stenosis obtained through tightening of the blood vessel.

These models are mainly used to understand the mechanism of thrombus development due to complete or partial blockage of blood flow, to study the interaction between platelets and coagulation factors, or to evaluate antithrombotic drugs.

Even though murine models are among the most used animal models in research, their main disadvantage in thrombosis research is their small size, making it challenging and complicated to perform intravascular interventions. However, they do provide the advantage of ease of maintenance with low cost. Also, the feasibility of genetic alternations helps elucidate the molecular mechanism and factors involved in this pathogenesis.

#### 4.2.2. Porcine Models

In addition to sharing more genetic similarities with humans than mice do [74], pigs also provide the advantage of large size, which means that thrombosis models resemble humans more closely than smaller species and vascular interventions can be employed more easily. Also, porcine hemodynamics, coagulation cascade, and the vascular system overall are closer to humans [75,76,77]. Considering their large size, porcine thrombosis models mostly involve surgical interventions and, thus, are considered more complicated and practically demanding in comparison to other smaller animal models.

##### Endothelial Dysfunction

In pigs, different methods can alter endothelial integrity and functions, thus promoting coagulopathies and thrombosis. One study reported that balloon angioplasty in carotid arteries promotes erosion of the endothelial cells at the inflation site, with some sites showing tears in the underlying layers and excessive platelet deposition, eventually leading to thrombus development with complete occlusion in some cases. Such models can be used to evaluate therapeutic options to prevent endothelial injury and vascular occlusion following angioplasty, which is considered a common consequence following this therapeutic procedure [78]. Other approaches involve the use of balloon angioplasty wrapped with metallic coil to facilitate endothelial injury upon balloon inflation [79]. Also, stents—whether bare metals or drug eluting stents—can be employed where they are implanted in the vessel and used as models to study restenosis and stent thrombosis that can occur following these surgical procedures, which limits their application [80].

Electrical stimulation can also induce endothelial injury and complete vascular occlusion without the need of vascular constriction. This phenomenon is believed to occur due to platelet deposition at the site of injury, thus making this model useful to compare between different antiplatelet drugs [81].

Other studies reported that pigs fed with a high-cholesterol diet for at least nine weeks display attenuated endothelial functions with non-responsiveness to serotonin or bradykinin in terms of vasorelaxation, which is a prominent sign of endothelial dysfunction [82,83].

##### Induction of Stasis or Stenosis

**Figure 5:**
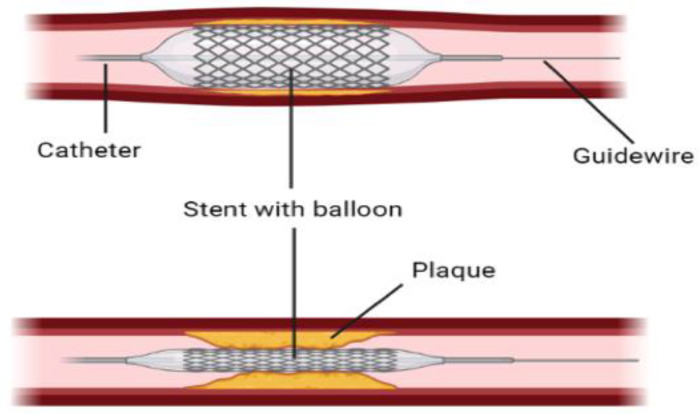
A graphical demonstration of stent with balloon angioplasty to open narrowed arteries. (Created with BioRender.com).

Balloon catheters or stent-based balloon catheters, which are normally used to dilate atherosclerotic blood vessels, as shown in Figure 5, are also used to limit blood flow or completely block it by controlling the degree of inflation. This method is employed in large animals like porcine models to study thrombosis development [84].

Surgical ligature or surgical placement of ameroid constrictors, which are rings that swell as they absorb the body fluid and gradually limit the blood flow, are also used to generate reproducible stenosis [85,86,87]. Different procedures that can limit the blood flow can be employed to develop thrombosis models, like vascular plugs or stents made from a variety of materials with different shapes and characters; these can be placed via inflatable balloons in different vascular areas to develop novel thrombosis models [88,89].

##### Promoting Coagulation

Thrombin-induced deep-vein thrombosis (DVT) is a common model used in thrombosis studies, where thrombin, which is a multifunctional enzyme, acts primarily as a pro-coagulant factor aiding in clot formation by converting fibrinogen into fibrin [90]. In this method, a vein is usually surgically exposed followed by local thrombin injection, and in some cases, stasis is created by narrowing proximal veins using different methods [91,92]. Thrombus formation is monitored and can be released by mechanical bending of the muscle or by saline infusion to reach different areas in the vasculature [87]. Thrombosis can be created in different areas using this technique. These models can be used to study the complex pathophysiology of thrombosis, evaluate anti-thrombotic drugs, and test thrombolytic devices like catheters.

A novel model of pulmonary hypertension (PH) with vascular remodeling that manifests as thrombosis was developed employing stenosis, and devoid of the complex surgical procedures employed in different PH models. In this model, a combination of distal embolization using dextran microspheres that were infused weekly to the pulmonary artery through a catheter and coiling of pulmonary branches using silk sutures was employed over a four-week duration alternating between left and right pulmonary arteries. This model was used to develop a chronic PH phenotype that recapitulates the human pathophysiology, with obstructed vascular lumen and occlusions in the vascular arteries [93].

High-fat and high-cholesterol diets are also commonly used to induce arterial atherosclerosis, by activating platelets and clotting factors and reducing the anti-thrombotic properties of the endothelium [94,95,96]. One study reported that maintaining minipigs on high-fat/high-sucrose diets for 6 months resulted in the development of fatty aortic lesions, which can be used as a model for diabetes-accelerated atherosclerosis to study the mechanism underlying this condition [97]. Another study reported that feeding miniature swine with a high-fat/high-cholesterol diet for 10 to 12 months resulted in accelerated atherosclerosis in different arterial sites, with some developing occlusive thrombosis [98].

##### Ex Vivo Arteriovenous (AV) Shunt Model

**Figure 6:**
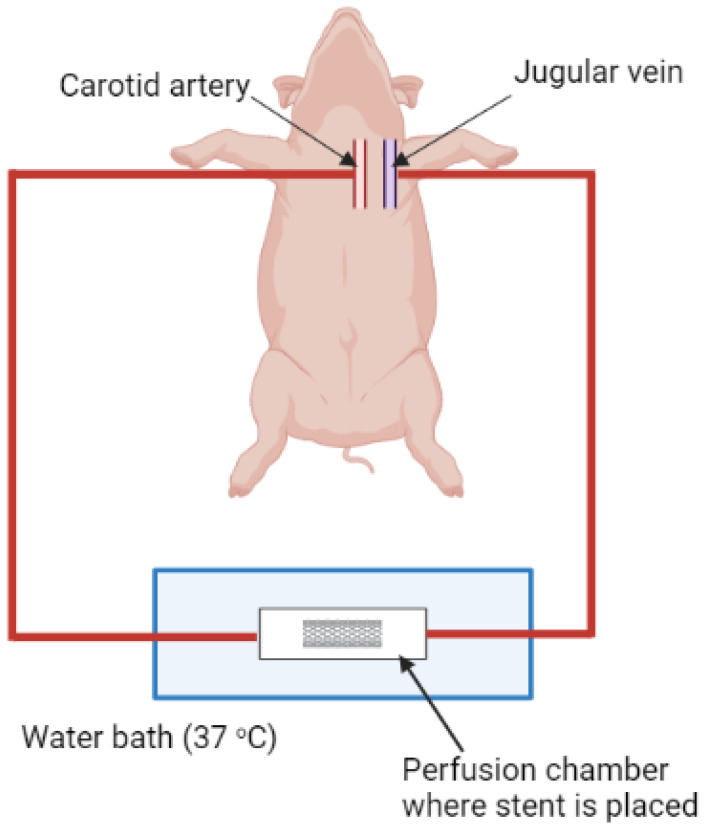
A diagram of porcine AV shunt model (Created with BioRender.com).

This is a more complicated but commonly used model that combines ex vivo and in vivo approaches. This model employs an extracorporeal perfusion system utilizing stents made from different materials and different morphologies that alter the shear rate of blood flow where the thrombus is developed. Figure 6 shows an illustration of the extracorporeal perfusion system, where stents can be placed in a perfusion chamber inside a water bath and an extracorporeal circuit is surgically utilized by connecting the carotid artery and jugular vein to external tubes, allowing the blood to pass through the perfusion chamber and return back to the jugular vein. Blood flow can be controlled and monitored using a pump and a flow meter connected to the extracorporeal circuit [99] (not shown in the figure).

The main disadvantages of using porcine models in research are related to their large size, which requires larger facilities for maintenance and, thus, higher costs. Also, handling these animals requires well-trained researchers to perform tests and surgical interventions. Ethical concerns are another aspect to be considered, as there are more restrictions when dealing with larger animals compared to smaller animals, like rats, mice, and zebrafish.

#### 4.2.3. Zebrafish

Zebrafish (*Danio rerio*) provide several advantages as model organisms, including their high reproduction rate, external fertilization, which allows ease of manipulation, and their transparency, which provides many advantages in labeling and visualization. Different human disorders have been modeled in zebrafish, including thrombosis.

Considering the small size of zebrafish, no methods involve surgical interventions or administration of an external device to induce stasis or stenosis. Most methods involve the use of chemicals, laser irradiation, or genetic alternations, as mentioned in detail below.

##### Induction of Endothelial Injury

As in murine models, this can be done by different techniques, where the most common is treatment with phenylhydrazine (PHZ), FeCl_3_, or laser irradiation. PHZ acts by generating superoxide radicals, causing damage to red blood cells through membrane lipid peroxidation, and enhancing thrombin generation [100]. The agent is also responsible for endothelial dysfunction leading to hypercoagulation, which all together act in thrombosis development [101]. Treatment of zebrafish 2 days post-fertilization with 1.5µM PHZ for 24 h developed a thrombosis model that was effective for screening of antithrombotic drugs [102].

FeCl_3_ and pulsed nitrogen laser irradiation were shown to promote vascular occlusion through endothelial injury, where FeCl_3_ causes damage throughout the vasculature, eventually forming a thrombus, while laser irradiation generates more of a local injury. Both models were effectively employed in genetic screening to identify genetic mutations involved in thrombosis [103].

##### Promoting a Hypercoagulable State

HFD can be used in zebrafish to induce hyperlipidemia and promote coagulation. A study showed that cholesterol appeared in zebrafish blood vessels on day five following a diet containing 8% cholesterol, and with time this developed into plaque, containing lipids and fibers [104]. Other parameters were also evaluated in this study and found to be consistent with human pathology of atherosclerosis (AS), where treatment with statin managed to effectively alleviate the symptoms. Thus, this approach can be used to model human AS and screen anti-AS drugs.

Genetic alternations, like inducing apoc2^−/−^ mutation and liver X receptor deletion, are other methods used in zebrafish to develop dyslipidemia models and promote coagulation for various applications [105,106].

Zebrafish models provide several advantages over the other in vivo models, including the ease of thrombosis production with high reproducibility, as no method requires surgical interventions, and thrombus development can be monitored in real time. The combination of simplicity and efficiency makes them good choices for thrombosis research.

Table 2 shows a list of some in vivo models of thrombosis. Other in vivo models, like dogs, rabbits, and primates, will not be included in this review; however, developing thrombosis in these models usually employs the same methods used in porcine and murine models.

### 4.3. Advantages and Disadvantages of In Vitro and In Vivo Models

#### 4.3.1. In Vitro Models

Different cells grown on a dish do not represent the whole physiological system with all its complexity and interaction with different components, though they usually provide a general estimate of what might happen inside the body, which needs to be further confirmed through the use of a full organism. A major argument against the use of microfluidics is the microchannels themselves, which, despite providing the advantage of using smaller blood volume, do not represent thrombosis that usually develops in larger arteries and veins [126]. Considering these, it is important to consider in vivo models for confirmation of results obtained from in vitro studies.

#### 4.3.2. In Vivo Models

Generally, no animal model can perfectly recapitulate any human disease, though their use has greatly advanced our understanding of human disorders and drug screening and development; thus, their use will continue to be central in research. Each animal model has its own limitations, where the choice will depend on several factors, including the aim of the study and availability of resources.

A brief comparison regarding the use of in vivo and in vitro models in thrombosis research are mentioned in the Table 3 below.

## 5. Conclusions

Approaches to generate thrombosis models in vivo and in vitro are wide and versatile, with many studies continuously developing novel techniques. Generally, methods that promote endothelial damage and activation of the coagulation pathway are used to induce venous thrombosis, while methods that promote platelet aggregation are used to induce arterial thrombosis. However, similar methods can sometimes be used to generate venous or arterial thrombosis, depending on the vessel type.

It should be noted that different methods generate thrombosis via different mechanisms and with different pathophysiology, as some studies have shown that thrombosis generated by different techniques responds differently to anti-thrombotic drugs. Thus, it is important to shift attention to standardize thrombosis models for each specific application, as there are large numbers of studies developing thrombosis models without specific and well-detailed characterization of the generated thrombus. Having a standard, reproducible model for each application can eliminate many of the ambiguities and non-consistent results in this field.

## Figures and Tables

**Table 1:** A list of some in vitro thrombosis models.

Type of In Vitro Model	Application	Reference
Parallel-plate flow chamber with endothelial cells matrix-covered surface	Compare various low-molecular-weight heparin and a pentasaccharide for suitability in the in vitro thrombosis model	[43]
Parallel-plate flow chamber-based model with fibrin- or fibrinogen-coated surface	Compare and characterize platelet adhesion to fibrin- and fibrinogen-coated surfaces under controlled flow	[26]
Parallel-plate flow chamber-based model with collagen- or plaque-coated surface	Compare the thrombogenic effect of different collagen fibers to atherosclerotic plaque	[30]
Flow chamber-based model with fibrinogen- or vWF-coated surface	Identify the mechanism of platelet adhesion to fibrinogen and vWF	[27]
Flow chamber-based model with collagen-coated surface	Identify the role of human collagen receptors GPVI and α2β1 in thrombus formation	[29]
Fibrinogen-coated flow chambers	Assess platelet adhesion and aggregation following incubation with H_2_-rich saline	[44]
Microfluidic-based device with blood flow under pathophysiological shear rate	Measurement of coagulation and platelet function	[34]
Microfluidic-based device with collagen-coated glass substrate	Measurement of platelet adhesion and blood viscosity	[35]
Microfluidic lung chip device lined with primary human alveolar epithelium	Monitor pulmonary thrombosis development and evaluate the effect of different pro-thrombotic and anti-thrombotic factors	[45]
Microfluidic device mimicking human venous valves	Develop a venous valvular stasis model and study the effect of platelets and red blood cells on thrombus development	[39]
Occlusive thrombosis-on-a-chip microfluidic device	Evaluation of anti-thrombotic drugs	[33]
Collagen-coated capillary with controlled rheological conditions	Examine the role of thrombin in platelet recruitment and thrombus stabilization	[46]
Collagen-coated glass stenosis model	Describe the structure of arterial thrombi	[47]
Endothelialized microfluidic device	Study the mechanism of FeCl_3_-induced thrombosis	[48]
Endothelialized microfluidic device	Study the effect of microplastics on thrombus properties	[49]
Endothelialized microfluidic device	A bioassay for hematological disorders and evaluating drug efficacy	[32]
In vitro human plasma clot formation assay	Compare the effect of aprotinin and tranexamic acid on the coagulation pathway and thrombus formation	[50]
3D-bioprinted thrombosis on a chip model coated with human endothelium embedded in a hydrogel	Develop a highly human biomimetic thrombosis model and study its pathophysiology and potential drug efficacy assessment	[51]
3D-printed microfluidic chip coated with human umbilical vein endothelial cells	Recapitulate the three-dimensional structure of healthy and stenotic coronary arteries and assess platelet aggregation	[52]
Annular and rectangular perfusion chambers with steady flow	Study the effect of endothelial cells activation on thrombus formation	[53]
Multiplate aggregometer and platelet function analyzer (PFA-100)	Test platelet aggregation to investigate cilostazol’s anti-platelet effect	[54]
Blood-shearing device	Study the influence of non-physiological stress on platelets and vWF	[42]

**Table 2:** A list of some common animal thrombosis models.

Method Employed in the Thrombosis Model	Mechanism of Thrombus Development	Application	Reference
**Porcine**			
Balloon angioplasty-induced thrombosis	Endothelial injury	Evaluate angioplasty-induced thrombosis	[78]
Angioplasty balloon wrapped with a metallic wire coil	Endothelial injury	Determine the relationship between the degree of vascular injury and restenosis magnitude	[79]
Surgical ligation and thrombin administration followed by thrombus release to induce PE	Stasis and promoting a hypercoagulable state	Develop a new venous thromboembolism model for possible use in therapeutic testing	[87]
Surgical ligation and thrombin administration	Stasis and promoting a hypercoagulable state	Develop a new model of chronic venous thrombosis	[92]
Balloon catheter and thrombin administration	Stasis and promoting a hypercoagulable state	Monitor thrombolytic procedures with magnetic resonance imaging	[91]
Pulmonary artery embolization with dextran microspheres and surgical coiling of pulmonary branches	Stenosis	Develop a new model of chronic pulmonary hypertension with thrombosis	[93]
High-fat/high-sucrose diet-induced atherosclerosis	Promoting a hypercoagulable state	Develop a model of diabetic atherosclerosis	[97]
High-fat/high-cholesterol diet-induced atherosclerosis	Promoting a hypercoagulable state	Develop and characterize a diet-induced atherosclerosis model	[98]
Surgical ligation of femoral vein and thrombin administration	Stasis and promoting a hypercoagulable state	Develop a DVT model and assess changes in the femoral vein gene expression	[107]
Mechanical arterial injury in combination with stent placement followed by total occlusion	Endothelial injury and stasis	Characterize a stent thrombosis model	[108]
Balloon catheter and thrombin infusion	Stasis and promoting a hypercoagulable state	Evaluate a high-intensity ultrasound pulse (histotripsy) as a method of thrombolysis	[109]
Ischemia-reperfusion injured tissue model	Promoting a hypercoagulable state	Evaluate the role of fish oil in thrombosis development	[110]
Balloon catheter and thrombin infusion	Stasis and promoting a hypercoagulable state	Develop a survivable and reproducible iliocaval DVT model for possible use in therapeutic and imaging modalities’ evaluation	[84]
Electrical stimulation of the carotid artery endothelium	Endothelial injury	Compare the effect of cilostazol to ticlopidine in inhibiting occlusive thrombus formation	[81]
AV shunt model with nitinol stent exposed to arterial blood under high shear rate	Altering blood flow	Evaluate the effect of aspirin, clopidogrel, and combined therapy in inhibiting stent thrombosis development	[99]
AV shunt model	Altering blood flow	Compare the thrombogenicity of nitinol to stainless steel stents	[111]
Balloon catheter occlusion	Stasis	Evaluate oral administration of low-molecular-weight heparin with a carrier compound in DVT treatment	[112]
Self-expanding stent-graft device	Altering blood flow through stasis	Evaluate a thrombolytic therapy with urokinase	[88]
Balloon catheter injury	Endothelial injury	Study the effect of ionizing radiation on thrombosis development	[113]
Balloon catheter occlusion	Stasis	Use computed tomography to identify lung perfusion abnormalities	[114]
Balloon catheter occlusion and thrombin administration	Stasis and promoting a hypercoagulable state	Evaluate the safety and efficacy of microtripsy thrombolysis treatment	[115]
AV shunt model	Altering blood flow	Study the effect of rivaroxaban alone or in combination with dual antiplatelet therapy	[116]
**Murine models**			
Laser-induced thrombosis in mice	Endothelial injury	Evaluation of anti-thrombotic drugs	[58]
Serum-induced thrombosis in rats	Promoting a hypercoagulable state	Compare the thrombogenicity of homologous and heterologous serum	[65]
Tissue factor-induced thrombosis in rats	Promoting a hypercoagulable state	Compare the anti-thrombotic effect of thrombin inhibitor and factor Xa inhibitor	[67]
Vascular ligation in mice	Stasis	Evaluate the influence of aging on thrombus resolution	[73]
FeCl_3_-induced thrombosis in mice	Endothelial injury	Develop a refined ferric chloride-induced thrombosis model and test it against anticoagulants	[63]
FeCl_3_ and laser-induced thrombosis in mice	Endothelial injury	Evaluate the potency and safety of anfibatide as an antithrombotic agent	[117]
Hypoxia-induced thrombosis in mice	Promoting a hypercoagulable state	Develop and study the mechanism of hypoxia-induced thrombosis	[118]
Thrombin-induced thrombosis in rats	Promoting a hypercoagulable state	Develop and characterize a thrombotic ischemia model that mimics human thromboembolic stroke	[119]
FeCl_3_-induced thrombosis in rats	Endothelial injury	Characterize the thrombus, evaluate a novel antithrombotic agent, and determine the relationship between vessel temperature and vascular occlusion	[62]
FeCl_3_-induced thrombosis in rats	Endothelial injury	Assessment of tiplaxtinin antithrombotic effect	[120]
Vascular ligation in rats	Stasis	Study the antithrombotic effect of grape seed proanthocyanidins extract	[121]
**Zebrafish**			
PHZ-induced thrombosis	Endothelial injury and promoting a hypercoagulable state	Assessment of antithrombotic drugs	[102]
PHZ-induced thrombosis	Endothelial injury and promoting a hypercoagulable state	Evaluate the antithrombotic effect of *Rubia cordifolia*	[122]
FeCl_3_ or laser irradiation-induced thrombosis	Vascular injury	Genetic screening	[103]
Arachidonic acid-induced thrombosis	Promoting platelet aggregation	Evaluate the antithrombotic effect of danhong injection	[123]
Arachidonic acid-induced thrombosis	Promoting platelet aggregation	Evaluate the antithrombotic effect of Wuliangye Baijiu	[124]
*Apoc*2 mutant zebrafish	Promoting a hypercoagulable state	Characterization of *apoc2* mutant zebrafish	[105]
*Heg*1 knockout zebrafish	Damaging the vascular endothelium integrity	Develop a zebrafish model of dilated cardiomyopathy and thrombosis and employ it in drug screening	[125]
High cholesterol and lipopolysaccharide diet	Promoting a hypercoagulable state	Drug screening	[104]

**Table 3:** A comparison between the advantages and disadvantages of in vitro and in vivo models in research.

Category	In Vitro Models	In Vivo Models
Reproducibility	Possible, especially when using the same device and conditions.	Variable, considering inter-species variations.
Ethical concerns	Minimal.	Strict, especially with larger animals.
Cost	Relatively cheap.	Relatively expensive.
Simplicity	Relatively simple, especially when using pre-designed devices.	Relatively complicated and time consuming.
Result translation	Results need to be further confirmed by in vivo studies.	Considering the settings, results can be more closely related to human conditions, with higher possibility of clinical translation.

## Abbreviations

**AV**	Arteriovenous
**AS**	Atherosclerosis
**CTEPH**	Chronic thromboembolic pulmonary hypertension
**EDTA**	Ethylenediaminetetraacetic acid
**DVT**	Deep-vein thrombosis
**HFD**	High-fat diet
**MI**	Myocardial infarction
**PHZ**	Phenylhydrazine
**PE**	Pulmonary embolism
**PH**	Pulmonary hypertension
**TM**	Thrombomodulin
**TF**	Tissue factor
**vWF**	von Willebrand factor
